# Dr. N. S. Davis to Dr. Q. C. Smith

**Published:** 1887-10

**Authors:** 


CHICAGO, ILL., SEPT. 1887.

65 RANDOLPH ST.

Q. C. SMITH, M. D.

DEAR DOCTOR,—I am not aware that it is customary for State Medical Societies to *assess any per capita tax* on the members of the County or Local Societies. Such a tax or "royalty" certainly could have *no legal* force, unless the State Society were *chartered by the Legislature*, and the *Charter* contained some *special* provision, giving the right to *grant Sub Charters to County Soeieties* and tax the members thereof, for certain specified purposes, and within specified limits. Yours truly,

Signed, N. S. DAVIS.

This is authority, on a subject which should receive careful consideration in all local Societies, and which should he brought up for final action at the next meeting of the Texas State Medical Association.

